# Reference based annotation with GeneMapper

**DOI:** 10.1186/gb-2006-7-4-r29

**Published:** 2006-04-05

**Authors:** Sourav Chatterji, Lior Pachter

**Affiliations:** 1 Department of Computer Science, University of California at Berkeley, Berkeley, CA, 94720, USA; 2 Department of Mathematics, University of California at Berkeley, Berkeley, CA 94720, USA

## Abstract

We introduce GeneMapper, a program for transferring annotations from a well annotated genome to other genomes. Drawing on high quality curated annotations, GeneMapper enables rapid and accurate annotation of newly sequenced genomes and is suitable for both finished and draft genomes. GeneMapper uses a profile based approach for mapping genes into multiple species, improving upon the standard pairwise approach. GeneMapper is freely available for academic use.

## Abstract 2

GeneMapper, a new program for transferring annotations from a well-annotated reference genome to other genomes, is described.

## Rationale

With large scale sequencing of vertebrate, fly, and worm genomes now underway, it is imperative to develop methods that produce high quality annotations of these newly sequenced genomes. Lack of genome wide, full length cDNA sequences for these species will make it virtually impossible to annotate these genomes completely using cDNA based methods such as Aceview [1]. An alternative approach is to transfer reference annotation from a well annotated genome (such as human and *Drosophila melanogaster*) to other (possibly draft) genomes. We call this 'reference based annotation'. In fact, annotation systems such as ENSEMBL [2] already incorporate reference based annotation as part of their gene prediction pipelines.

The rationale behind the reference based approach is that a lot of resources have been invested in annotating genomes of model organisms, and it is unreasonable to expect similar efforts to be expended for the myriad of genomes that are now being sequenced. The status of current annotation projects for various insect and chordate genomes is shown in Table 1. In the case of vertebrate genomes, the human genome provides an excellent source of reference annotations suitable for transfer. In addition to having extensive numbers of cDNA sequences and a fairly complete RefSeq gene annotation, the human genome annotation also consists of a manual annotation component. By contrast, the other vertebrate genomes have insufficient cDNA sequence. In fact, many genome projects lack sufficient resources to run some of the existing *ab initio *gene prediction programs. The reference based annotation tool we have developed, called GeneMapper, can be used in such cases to transfer human annotations. GeneMapper provides a comprehensive annotation that, as we show, is surprisingly accurate. A similar argument can be made for other clades. For example, *D. melanogaster *is an extensively studied model organism, and there is a well curated FlyBase database [3] of supporting annotations. GeneMapper has been used to provide high quality annotations of the newly sequenced fruitfly genomes by transferring the FlyBase annotations.

Existing computational gene finding methods can be broadly classified into two main categories: *ab initio *methods and evidence based methods. *Ab initio *gene finding methods such as GENSCAN [4] and GENIE [5] predict the gene structure from first principles without using external evidence. Comparative *ab initio *gene finding methods such as SLAM [6], Twinscan [7], and SGP-2 [8] use conservation of gene structure among related species, for example human and mouse, to derive more accurate predictions. They exploit the fact that coding exons are functional and therefore are more likely to be conserved than noncoding sequence. More recently, methods such as Shadower [9,10], GIBBS [11,12], EXONIPHY [13], and NSCAN [14] use conservation information among multiple species to make gene predictions.

Evidence based gene finding methods are considerably more accurate than *ab initio *methods because they rely on information that is not intrinsic to the genome to improve prediction. Such information, called external evidence, can be in the form of cDNA or protein sequences from other species. Use of such information frequently requires alignment programs. In the case of cDNA, in order to make use of the evidence, programs such as Aceview [1], ecGene [15], GMAP [16], and BLAT [17] align cDNA with genomic sequence. These methods need to account for the fact that expressed sequence tags can have a relatively high error rate (up to 3%). However, they have not been developed to project cDNA evidence onto distantly related species. For example, they are not designed to align human cDNA with the mouse genome.

Another class of evidence based methods makes use of alignments of protein sequences with genomic sequences, and form an important component of pipelines such as ENSEMBL. Such programs include DPS [18], Procrustes [19], GeneWise [20], and GenomeScan [21]. To some extent, these programs are designed to work with proteins from related species. Although they work quite well with highly conserved proteins, they are not as accurate for diverged protein sequences. Hybrid methods such as JIGSAW [22] and ExonHunter [23] combine both cDNA and protein evidence probabilistically while making gene predictions.

GeneMapper has been influenced by and is in the same category of gene finding methods as Projector [24]. Projector uses gene annotations from a reference species as evidence to predict the gene structure in a target sequence. In analogy to cDNA based methods, Projector aligns mRNA from a reference gene to a target sequence, but it exploits additional information about splice sites. This is accomplished by using a pair hidden Markov model to transfer annotations from the reference species to the target sequence.

GeneMapper uses a bottom up approach to predict gene structure. First, each reference exon is aligned to a target genome and these alignments are then joined to build a gene structure. Because exons are much shorter than introns, this approach makes use of dynamic programming with a fairly sophisticated codon evolution model to provide detailed alignment of exons. GeneMapper also uses a novel mapping process that exploits the phylogeny of the reference and target species to obtain more precise annotations. If a gene is to be mapped from a reference species to multiple target species, then GeneMapper makes use of characteristic properties extracted from all of the available orthologous genes in the family. In other words, the program works with profiles of orthologous genes, which are not unlike protein profiles. The gene profile is built up progressively as the gene is mapped into successive target species. Therefore, the profile becomes more complete as the gene is mapped into additional target species. The profile is especially useful in mapping genes to evolutionarily distant species that may have diverged considerably from the reference species. The rationale behind the profile based approach is that information from all orthologous sequences results in a more comprehensive representation of the gene than is possible with a single sequence.

GeneMapper was tested on a set of orthologous human and mouse genes. Results were compared with GeneWise and Projector annotations. We show that GeneMapper outperforms both GeneWise and Projector, and also establish that the addition of multiple sequences from chimpanzee, rat, and chicken further improves performance through the use of gene profiles.

## Results

GeneMapper was implemented in the computer programming language C and tested on a standard Linux machine. The running time of GeneMapper on a single gene is given by the following equation:



where N_e _is the number of exons in the gene and l_i _is the length of the ith exon. A loose upper bound on this running time is O(L^2^), where L is the length of coding sequence in the gene. However, the running time is expected to be appreciably smaller than quadratic for multiple exon genes. GeneMapper can be downloaded from the GeneMapper website [25].

Two tests were conducted to evaluate the performance of GeneMapper. In the first test, GeneMapper was compared with GeneWise and Projector, two commonly used reference based programs. For the second test, a data set of orthologous genes from the human, chimpanzee, mouse, rat, and chicken genomes was created. This data set was then used to test the hypothesis that adding more species improves the performance of GeneMapper. The tests are described in detail in the following two sections. Finally, GeneMapper was used to annotate ENCODE [26] regions by transferring human GENCODE [27] annotations to other species. We believe that this data set will be an important resource for studying the evolution of genes in vertebrate genomes.

### Performance

GeneMapper was compared with Projector and GeneWise on the Projector data set [24]. This data set consists of 491 orthologous genes that are reciprocal best matches between mRNA supported human and mouse ENSEMBL genes. The set can be divided into two subsets. The first subset contains 465 genes for which the number of exons is the same in the human and mouse orthologs. The second subset has 26 genes in which the human and mouse orthologs have different number of exons, in some cases resulting from exon fusion and splitting events. Some of the genes in this subset were not true orthologs and the data set was refined manually to remove any such errors. The refined data are in Additional data file 1.

To compare the performance of the programs, the human annotations were used to predict the gene structure in the orthologous mouse sequences. GeneWise and Projector predictions were taken from the Projector paper [24]. The eval package [28] was then used to calculate the nucleotide, exon, and gene level sensitivities and specificities of the programs. For more details about these metrics, the reader is referred to the report by Burset and Guigo [29]. The performances of the three programs are compared in Table 2. The exon level sensitivity and specificity of GeneMapper is 97.15% and 98.19%, respectively, and the error rate is less than half that in the other programs. The gene level sensitivity and specificity is improved by more than 20% compared to GeneWise and Projector. We believe that the primary reason for GeneMapper's accuracy is the use of a proper exon model for the alignment and mapping of exons. The results clearly indicate that GeneMapper represents a significant improvement over existing programs and will be a useful tool for accurately transferring annotations from reference genomes to the newly sequenced genomes.

### Using additional species to improve performance

The second test used a data set of orthologous human, chimpanzee, mouse, rat, and chicken genes to measure the improvement in accuracy of GeneMapper with the addition of multiple species. RefSeq annotations of human, mouse, and chicken genomes were downloaded from the University of California Santa Cruz (UCSC) genome browser database [30]. The gene set was refined to remove annotations with common errors such as the absence of start or stop codons. BLAT [17] was then used to find mutually best hits among the proteomes. The pair-wise hits were further joined together to obtain orthologous triplets of human, mouse, and chicken genes. The human and mouse orthologs were then mapped into the chimpanzee and rat genomes, respectively, resulting in a set of orthologs from all five species. The data set obtained by this process consisted of 895 potential orthologous segments from the five vertebrate genomes, and is provided in Additional data file 2. We should note here that this standard method of obtaining orthologs by reciprocal best hits cannot distinguish between paralogs. However, the accuracy of reference based programs such as GeneMapper is not affected as long as the potential orthologs are sufficiently conserved.

To assess the performance of pair-wise GeneMapper, human annotations were used to predict the gene structure in the orthologous chicken sequences. For the multiple species version of GeneMapper, additional orthologous sequences from chimpanzee, mouse, and rat were utilized. The profiles were initialized with the human genes, and were then used to predict gene structures incrementally in the chimpanzee, mouse, and rat genomes. As gene structures were predicted in each new species, they were added to the profiles. Finally, the profiles were used to predict the gene structures in the chicken sequence. The performance of the pair-wise and multiple species versions of GeneMapper on the chicken genome is summarized in Table 3. The Table demonstrates that multiple species GeneMapper represents an improvement over pair-wise GeneMapper. We point out below that most of the errors in the predictions are caused by factors that cannot be corrected computationally. Consequently, it is quite significant that multiple species GeneMapper is able to correct 18 wrong exon predictions of pair-wise GeneMapper with just three additional species. We therefore believe that, with the addition of more species, multiple species GeneMapper will come close to the limit of computational reference based methods.

### ENCODE annotations

The goal of the ENCODE project [26] is to study functional elements by rigorously analyzing a portion (about 1%) of the human genome. Forty-four regions across the human genome were chosen for investigation and orthologous regions in other vertebrate genomes were sequenced for comparative analysis. GeneMapper was used to annotate the ENCODE regions by transferring human GENCODE [27] annotations to other species. We provide these annotations as a resource for studying the evolution of genes (Additional data file 3).

## Discussion

We have shown that GeneMapper can transfer reference annotations with remarkably high accuracy and that it is a substantial improvement over existing programs. This suggests that reference based gene finding is a feasible approach for accurately annotating the large number of genomes that are now being sequenced.

It is important to note that the concept of transferring annotations is not a new one, and methods such as DPS, Procrustes, GeneWise, Genomescan, and Projector have been designed to perform exactly the same task. GeneWise and Procrustes align proteins with genomic sequences from target species. The principal disadvantage of the protein alignment approach is that it does not utilize information about exon/intron boundaries and therefore does not perform very well on less conserved genes. On the other hand, methods such as Projector and GeneMapper utilize the exon/intron structure of the gene and thus are more accurate in identifying splice sites. However, it should be noted that GeneMapper and Projector are not suitable for mapping genes from very distant species, in which the exon/intron structure of the gene might not remain conserved. For example, if one wants to find the homolog of a novel fruitfly gene in the human genome, it is probably best to use methods such as Procrustes and GeneWise.

Both GeneMapper and Projector use the exon/intron structure of the gene to predict the ortholog of a reference gene in a related species, but they have different approaches to the prediction problem. Projector uses the Viterbi algorithm for a pair hidden Markov model to predict the gene structure. Because the running time of the Viterbi algorithms for pair hidden Markov models is quadratic, Projector uses a heuristic to decrease the search space. In contrast, GeneMapper uses a bottom up algorithm that first maps each exon and then joins the exon predictions together to obtain the gene structure. Because exons are much shorter than introns, a more sophisticated model can be used for exon alignment. The optimal alignment is still obtained using dynamic programming, albeit a more complex one. We believe that the use of our exon alignment model makes GeneMapper more accurate than Projector. Furthermore, unlike Projector, GeneMapper models sequencing errors and frameshifts, and we believe that this makes GeneMapper more suitable for draft genomes.

When a gene must be mapped into multiple species, GeneMapper uses profiles to derive a more complete characterization of the gene and thus make more precise predictions. This is because a profile of orthologous genes can help us to obtain much more information about the gene family than a single reference gene. We showed that the use of additional species and the application of the profile based approach outperforms the pair-wise approach. The use of profiles is particularly appropriate for annotating the newly sequenced vertebrate, insect, and worm genomes because the profile can exploit information from all related genomes while making gene predictions.

### Potential sources of error

Even though GeneMapper is remarkably accurate and has an error rate of less than 3% in transferring exons from human genes to orthologous mouse sequences, we investigated the sources of these errors to gain more insight into the GeneMapper algorithm. Most errors can be classified into the categories explained below.

Exons that have diverged considerably between the reference and the target genes are unable to pass the statistical significance tests of ExonAligner. This is because a choice was made to report only highly reliable predictions at the cost of missing a few true exons.

As described in the Methods section (below), GeneMapper's procedure for detecting exon splitting is comparatively crude and depends on accurate alignment of the reference exon with the orthologous target sequence (which contains an inserted intron). The presence of the inserted intron makes it difficult to align these regions accurately, especially if it is a long intron. Such wrongly aligned exons are partially predicted and this problem can probably be solved by employing a more sophisticated alignment model that allows inserted introns.

The GeneMapper algorithm is unable to account for certain assembly and sequencing errors. For example, we found many cases of duplicated chicken exons, most probably due to errors in the assembly. In such cases there is no way to distinguish between the duplicate exons, and the prediction is made randomly among the duplicates. GeneMapper also constrains the predicted exons to have splice sites at their ends. Therefore, we are unable to deal with sequencing errors at splice sites.

Differential splicing in the reference and target species can also cause errors in GeneMapper predictions. For example, if an exon is transcribed in the reference species but its ortholog is not transcribed in the target species, then GeneMapper predicts a wrong exon in the target species. However, it is not clear whether this is a wrong prediction, considering that this exon might be part of an alternate transcript in the target species. In fact, whether alternative spliced forms are conserved among related species such as human and mouse is an open question, and we believe that GeneMapper predictions could be an appropriate starting point for any experiment that seeks to address this issue.

An analysis of these errors will facilitate future improvements in GeneMapper. For example, we intend to work on statistical significance tests that are able to do a better job in discriminating between true and false exon predictions. Future enhancements of GeneMapper will also include improved handling of exon splitting. GeneMapper only transfers the coding sequence of a reference gene to a target sequence. We intend to modify GeneMapper to map 5' and 3' untranslated regions. This will also help in mapping short initial/terminal coding exons, which are more divergent compared with internal exons.

Although, as we point out, there is still room for improvement, we believe that multiple species GeneMapper comes close to the limit of gene prediction accuracy that is possible with computational reference based gene finding.

## Methods

### ExonAligner

GeneMapper is a bottom up algorithm that first predicts the ortholog of each reference exon in the target sequence and then combines the exon predictions to determine the gene structure. Therefore, the most critical step in the algorithm is to predict the ortholog of each reference exon by aligning it with the target sequence. A module called ExonAligner was developed to carry out this step in GeneMapper. ExonAligner takes as input two sequences, the annotated exon from the reference species and a target sequence containing its ortholog. A fairly intricate dynamic programming model is then used to align the reference exon with the target sequence.

**Figure 1:**
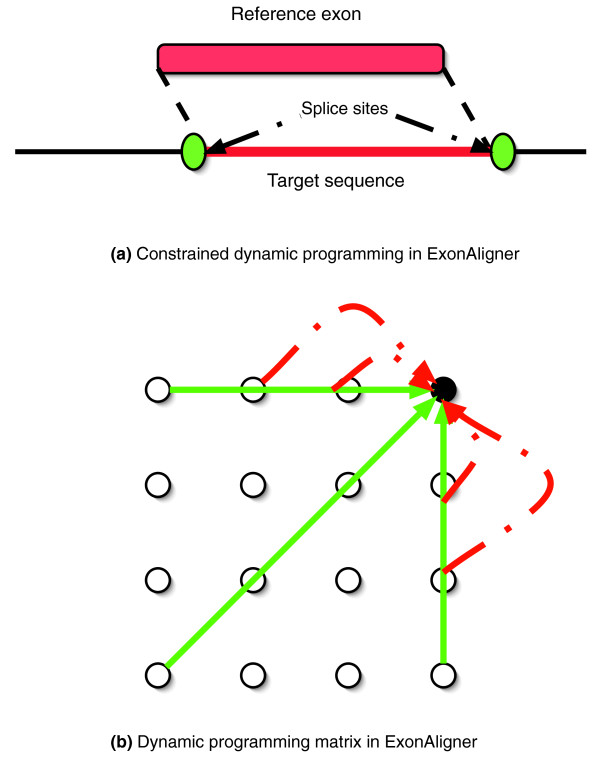
The ExonAligner algorithm. **(a) **Representation of constrained dynamic programming used by ExonAligner. It aligns the reference exon with a subsequence of the target sequence. This subsequence is additionally constrained to have splice sites at its ends, which are represented by green blobs in the cartoon. **(b) **The dynamic programming matrix used by ExonAligner. Only the edges into top right node are shown. The solid edges represent matches/mismatches and gaps in codon space. The dotted edges represent translation frame disrupting events such as frameshifts.

ExonAligner uses a version of the Smith Waterman algorithm to find the best alignment of the reference exon with a subsequence of the target sequence. In this version of the standard dynamic programming algorithm, as shown in Figure 1a, overhanging ends are penalized in the reference exon but not in the target sequence. In addition, the matched subsequence is constrained to have splice sites at its boundaries. The splice sites are scored using StrataSplice [31] to improve splice site detection.

ExonAligner uses a special dynamic programming matrix to model the evolution of codons and to allow for sequencing errors and frameshifts. The dynamic programming matrix is shown in Figure 1b. There are two types of edges in the matrix, with solid edges representing transitions in codon space and dotted edges representing events that cause disruptions in the translation frame. The solid edges model insertions, deletions and pairing of codons, and cover three nucleotides in the X and/or Y coordinates. On the other hand, the dotted edges cover one nucleotide in the X or Y direction. They model events such as sequencing errors and frameshifts, which cause disruptions in the translation frame. Because these events are very rare, a large penalty is charged for traversing these edges.

ExonAligner models the evolution of codons by using 64 × 64 COD matrices. COD matrices are very similar to PAM and BLOSUM matrices [32,33], which define distances between amino acids. The COD matrices are learned from whole genome alignments. In the case of vertebrates, the COD matrices are extrapolated from human and chimpanzee whole genome alignments. The whole genome alignment of the human and chimpanzee genomes was obtained from the UCSC genome browser database [30]. The alignments of human genes with the chimpanzee genome were extracted from these data. The gene alignments were then used to learn parameters for evolution of codons between human and chimpanzee genomes. The human/chimpanzee parameters were extrapolated to obtain parameters for other species.

The ExonAligner algorithm predicts the reference exon's putative ortholog in the target species. The putative ortholog is used as a prediction by GeneMapper only if its alignment with the reference exon passes a test of statistical significance. The testing of statistical significance of alignments is a well studied problem. The reader is referred to the book by Durbin and coworkers [34] for an overview. ExonAligner uses the Bayesian likelihood ratio test as its core test. In this test, the calculated score is the ratio of the likelihood of the alignment in the match model to its likelihood in the random model. Because the score is dependent upon length, short exons may fail to pass the ratio test. Therefore, ExonAligner also allows highly conserved short exons to pass the test of statistical significance.

### The pair-wise GeneMapper algorithm

**Figure 2:**
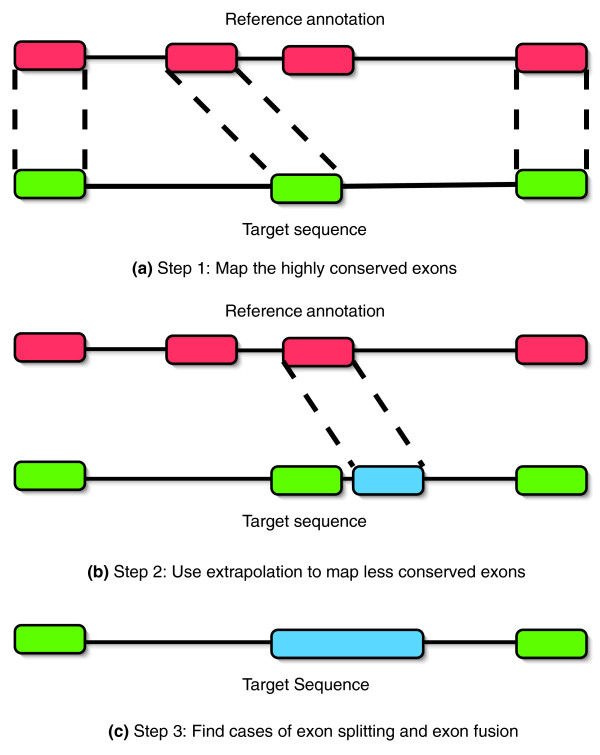
The three stages of the GeneMapper pipeline. **(a) **The first stage, in which only the most conserved exons are mapped. **(b) **The second stage, in which the algorithm uses exons mapped in the first stage as signposts to map already mapped exons. In this example, the possible locations of the second and third exons are narrowed down because they must be between the first and fourth exons. **(c) **The last stage, in which the algorithm searches for cases of exon splitting and exon fusion.

In this section we describe the pair-wise version of GeneMapper, which maps gene annotations from a reference species to a single target species. The GeneMapper pipeline consists of three stages, shown in Figure 2. In the first stage only the most conserved exons are mapped to the target sequence. At the end of this stage, an approximate outline of the gene in target sequence is obtained, as shown in Figure 2a. In the second stage this outline is used to predict the orthologs of exons that are unmapped in the first stage. The exons mapped in the first stage narrow down the possible locations of neighboring unmapped exons and thus help in mapping them with more confidence. For example, in Figure 2b the search for the third exon in the target sequence can be narrowed down between the second and fourth exons (which were mapped in the first stage of the algorithm). In the first two stages, it is assumed that there are equal numbers of exons in orthologous genes of the reference and target species. However, studies [35] have shown that this is not entirely true. In case of human and mouse, for instance, about 15% of orthologous genes do not have the same number of exons. Therefore, GeneMapper searches for exon splitting and exon fusion events in the third stage. We now describe in detail each stage of the pipeline.

In the first stage of the GeneMapper algorithm, only the highly conserved exons are mapped. GeneMapper initially searches for the approximate locations of the ortholog of each exon in the target sequence by using translated BLAST. If any significant hits are found for an exon, then the best hit is extended to derive an approximate location of the exon's ortholog in the target sequence. The ExonAligner algorithm is then used to predict the exact ortholog of the exon. The alignment of the predicted ortholog with the reference exon is checked for statistical significance using a combination of tests (described above). These tests are made quite stringent so that only the most conserved exons may pass them. This choice is made by design because we are able to obtain an outline of the gene structure in the target sequence that can be utilized to map less conserved exons more confidently in the next stage of the algorithm.

**Figure 3:**
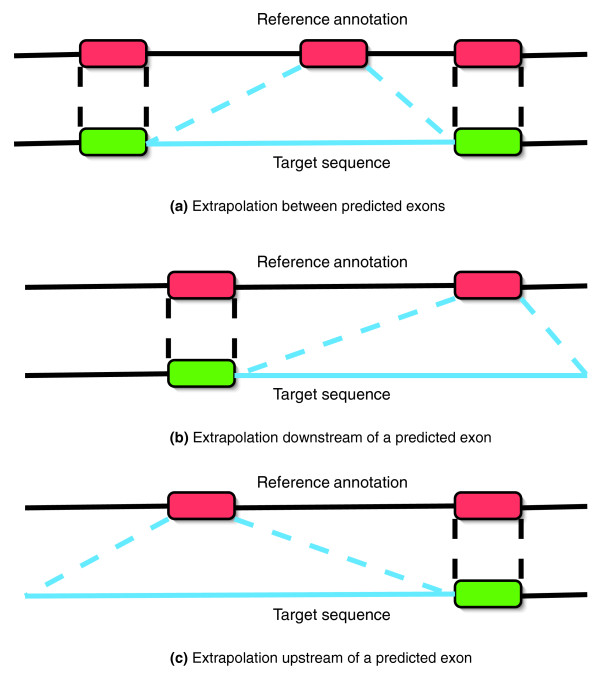
Extrapolation in GeneMapper. Use of extrapolation to pinpoint the location of unmapped exons in the second stage of GeneMapper pipeline. The blue sequence shows the possible location of the unmapped exon in the target sequence, and we assume that the gene is in the same strand in both species. **(a) **If an unmapped exon has mapped exons both to its upstream as well as downstream, then the unmapped exon should be mapped between the orthologs of its nearest mapped upstream and downstream exons. **(b) **If only the exons upstream of an unmapped exon are mapped, then the unmapped exon should be mapped downstream of the ortholog of its closest mapped exon. **(c) **If only the exons downstream of an unmapped exon are mapped, then the unmapped exon should be mapped upstream of the ortholog of its closest mapped exon.

In the second stage of GeneMapper, linearity of transcription is used to map exons that are missed in the first stage of the algorithm (specifically, already mapped exons are used to find out the approximate locations of unmapped exons). The details of the use of extrapolation to pinpoint the location of unmapped exons is shown in Figure 3. Once the possible location of an unmapped exon has been narrowed down, translated BLAST and ExonAligner are used to map the exon in the target sequence by a procedure that is similar to the first stage of the algorithm. However, the statistical significance tests are made less stringent in the second stage. This is because the position of the exon was narrowed down using already predicted exons, and this makes us more confident about the accuracy of the prediction.

In the third and final stage of GeneMapper, the algorithm searches for exon fusion and exon splitting events. For detecting exon fusion, we exploit the fact that introns must be of a minimum length to maintain the intron splicing reaction. Thus, if two adjacent exon predictions in the target sequence are closer than the minimum intron length, then they must have fused during evolution. This rule is very effective in detecting most cases of exon fusion in the Projector data set. On the other hand, the rule for detecting exon splitting is comparatively crude and is dependent on having an accurate alignment of the reference exon with the predicted ortholog. The alignment is searched for gaps of length greater than the minimum intron length and having splice sites at their ends. Such gaps are best explained by exon splitting events. The rules for detecting exon splitting are preliminary and improvements are planned in future versions of GeneMapper.

### Multiple species GeneMapper

Several studies [11,14,36,37] have shown that increasing the number of species helps in improving the performance of comparative *ab initio *gene finding programs. It therefore appears intuitive that increasing the number of species (and thus increasing the amount of available data) should enhance the accuracy of evidence based gene finding methods. The multiple species version of the GeneMapper algorithm makes use of two key ideas to improve upon the pair-wise algorithm. First, a profile of the gene is built and updated each time we map the gene into a new target species. The gene profiles are very similar to protein profiles, which are used extensively in protein informatics. The profiles help us to map genes more accurately into species that are evolutionarily distant from the reference species. Second, there is a specific order in which a gene is mapped from the reference species into the multiple target species, and this order is designed to take full advantage of the profile.

**Figure 4:**
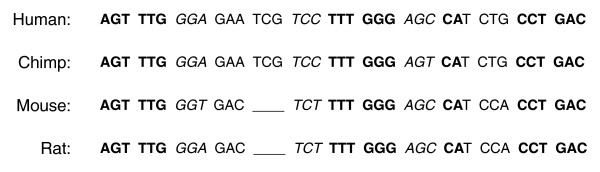
A gene profile. A portion of the gene profile of the *Neurod4 *gene orthologs in human, chimpanzee, mouse, and rat. Each column in the profile contains orthologous codons and is used to obtain the residue scoring matrix for dynamic programming. Columns with conserved codons are shown in bold, whereas columns with synonymous substitutions are italicized.

Gene profiles are alignments of one or more orthologous genes that are used to search for new orthologs. As shown in Figure 4, gene profiles work in codon space and each column in the profile contains orthologous codons. As with standard profiles, a gene profile can include gaps of length 3 that cover a codon. For example, the fifth column in the figure has codon gaps in the mouse and rat sequences. In addition, a gene profile can contain noncodon gaps that cover one nucleotide. These gaps account for rare translation disrupting events such as frameshifts and sequencing errors and are not shown in the Figure.

ExonAligner is modified to align gene profiles with sequences. As with pair-wise ExonAligner, COD matrices are used to model the evolution of codons. To evaluate the residue scoring matrix for the profile, ExonAligner calculates the COD matrices defining the distances between the codons in the target species and each species in the profile. The COD matrices are then used to derive the pair-wise residue scoring matrix for each species. The residue scoring matrix for the whole profile is the sum of the pair-wise scores. We illustrate the procedure by calculating the residue scoring matrix for species s at the third column in Figure 4. We first calculate the pair-wise COD matrices between species s and human, chimpanzee, mouse and rat, and call them COD_sh_, COD_sc_, COD_sm _and COD_sr_, respectively. The score for codon c is sum of the pair-wise scores:

COD_sh_(c, GGA) + COD_sc_(c, GGA) + COD_sm_(c, GGT) + COD_sr_(c, GGA)

ExonAligner uses two evolutionary models to take into account the variations in mutability of codons. The first model represents codons that are under negative selection and have low mutation rate. The second model represents codons that are not under any selection pressure and therefore have a high rate of mutability. A simple heuristic is employed to determine the model for a particular site. The first model is used if all of the mutations in the site are synonymous; otherwise, the second model is used. In addition, the program uses position sensitive gap scores, whereby sites represented by the second model have a lower gap penalty.

The mapping of the gene into each target species takes place in three stages, in exactly the same manner as for pair-wise GeneMapper (see above). The sequence in which the target species are mapped is ordered by the evolutionary distance from the reference species; specifically, the gene is first mapped to the target species closest to the reference species, then to the next closest species, and so on. This particular order is used because it is comparatively easier to map genes to a species that is evolutionarily close to the reference species than to a species that is more distant. Each time an orthologous gene is predicted in a target species, it is added to the profile. The updated profile is a more complete representation of the statistical properties of the gene family and therefore helps us to derive a more accurate prediction of the ortholog in the next species.

## Additional data files

The following additional data are included with the online version of this article: a gunzipped tar file containing the data set of orthologous genes in human and mouse that was used to compare GeneMapper with Projector and GeneWise (Additional data file 1); a gunzipped tar file containing the data set of orthologous genes in five vertebrates (human, chimpanzee, mouse, rat and chicken) that was used to compare pair-wise and multiple species GeneMapper (Additional data file 2); and a gunzipped tar file containing GeneMapper annotations of the ENCODE regions (Additional data file 3).

## Supplementary Material

Additional data file 1 A gunzipped tar file containing the data set of orthologous genes in human and mouse that was used to compare GeneMapper with Projector and GeneWise Click here for file

Additional data file 2 A gunzipped tar file containing the data set of orthologous genes in five vertebrates (human, chimpanzee, mouse, rat and chicken) that was used to compare pair-wise and multiple species GeneMapper Click here for file

Additional data file 3 A gunzipped tar file containing GeneMapper annotations of the ENCODE regions Click here for file

## Figures and Tables

**Table 1:** Annotation status of vertebrate and fly genomes

Organism	EST sequences	Genbank mRNA	RefSeq genes	Manual annotations	*Ab initio *tracks
*Homo sapiens*	6,134,812	207,905	24,293	22,421	5
*Pan troglodytes*	4,983	947	None	None	3
*Macaca mulatta*	52,754	1,766	None	None	None
*Canis familiaris*	349,306	1,666	None	45	2
*Bos taurus*	702,434	8,046	None	None	2
*Mus musculus*	4,686,082	241,865	18,757	5,501	3
*Rattus norvegicus*	701,072	23,017	9,012	None	5
*Oryctolagus cuniculus*	28,046	2,669	None	None	None
*Dasypus novemcinctus*	None	None	None	None	None
*Loxodonta africana*	None	4	None	None	None
*Monodelphis domestica*	50	363	None	None	1
*Gallus gallus*	578,445	29,743	3,848	None	4
*Xenopus tropicalis*	1,038,272	10,712	None	None	1
*Dana rerio*	673,076	25,094	10,689	3,546	None
*Tetraodon nigroviridis*	99	107,945	None	None	2
*Takifugu rubripes*	25,850	978	None	None	1
*Drosophila melanogaster*	383,407	19,931	19,697	None	4
*D. simulans*	5,013	80	None	None	2
*D. yakuba*	11,015	808	None	None	2
*D. erecta*	None	6	None	None	1
*D. ananassae*	None	11	None	None	1
*D. pseudoobscura*	35,042	40	None	None	4
*D. virilis*	663	41	None	None	1
*D. mojavensis*	361	2	None	None	1
*D. grimshawi*	None	None	None	None	1

The Table summarizes the annotation status of vertebrate and fly genomes as of October 2005. The numbers of expressed sequence tag (EST) sequences were obtained from the NCBI dbEST database [38]. The number of manually annotated genes was obtained from the VEGA annotation project site [39]. Other numbers were obtained from the UCSC genome browser database [30].

**Table 2:** Performance of reference based programs

Program	Nucleotide		Exon		Gene^a^	
	
	Sensitivity	Specificity	Sensitivity	Specificity	Sensitivity	Specificity
GeneWise	99.86	99.91	92.8	93.4	61.3	60.8
Projector	99.78	99.70	94.2	90.5	59.9	59.5
GeneMapper	99.88	99.94	97.2	97.8	81.7	81.7

The Table summarizes the performance of GeneWise, Projector and GeneMapper on the Projector data set consisting of 491 orthologous human and mouse genes. The human annotation was used to predict the gene structure in the mouse sequence. Performance is reported in terms of nucleotide, exon, and gene level sensitivities and specificities. ^a^GeneMapper predicts exactly one gene per reference annotation, and the number of predicted genes is equal to the number of genes in true or gold standard annotation. Consequently, gene sensitivity is equal to gene specificity for GeneMapper.

**Table 3:** Comparison of pairwise and multiple species GeneMapper

Program	Nucleotide		Exon		Gene	
	
	Sensitivity	Specificity	Sensitivity	Sensitivity	Specificity	Sensitivity
Pair-wise GeneMapper	99.95	99.93	91.3	95.1	52.2	52.2
Multiple species GeneMapper	99.95	99.93	91.5	95.2	52.6	52.6

The Table summarizes the effect of additional species on the performance of GeneMapper. To test pair-wise GeneMapper, only the human annotation was used to predict the gene structure in the chicken sequence. To test the profile based approach, additional orthologous sequences from the chimpanzee, mouse, and rat genomes were used to create a profile for each gene. The profiles were then employed to predict genes in the chicken sequences. The Table compares the accuracy in predicting the gene structure in the chicken sequences.
